# Special Issues Encountered When Cancer Patients Confront COVID-19

**DOI:** 10.3389/fonc.2020.01380

**Published:** 2020-08-07

**Authors:** Lina Qi, Kailai Wang, Chenyang Ye, Shu Zheng

**Affiliations:** ^1^School of Medicine, Cancer Institute (Key Laboratory of Cancer Prevention and Intervention, China National Ministry of Education), The Second Affiliated Hospital, Zhejiang University, Hangzhou, China; ^2^Research Center for Air Pollution and Health, School of Medicine, Zhejiang University, Hangzhou, China; ^3^Department of Surgical Oncology, The Second Affiliated Hospital, Zhejiang University School of Medicine, Hangzhou, China

**Keywords:** COVID-19, cancer, diagnosis, anticancer treatment, prognosis

## Abstract

Since the beginning of the COVID-19 global pandemic, there has been insufficient evidence and experience to help oncologists understand how to deal with infected and non-infected cancer patients. Many hospitals worldwide have shared their experiences of managing such patients by using the internet to reach non-infected cancer patients. However, for infected or suspected infected cancer patients, their experiences in terms COVID-19 diagnosis, anticancer treatment and prognosis are largely unknown and controversial. Here, we summarize the incidence, severe illness rate and mortality according to the published clinical data of COVID-19 in cancer patients and discuss the diagnostic difficulties, anticancer treatment and prognosis of COVID-19-infected cancer patients.

## Introduction

Since the outbreak of COVID-19 in December 2019, the resulting pandemic has overwhelmed medical care facilities worldwide and attracted tremendous public and social media attention. In such conditions, it is difficult for cancer patients to obtain timely and high-quality medical services as is usual due to social distancing and the utilization of medical resources for the COVID-19 crisis. A large number of cancer patients are subject to a high COVID-19 infection risk due to decreased immunity caused by cancer itself, anticancer treatments, such as chemotherapy, radiotherapy or surgery (1) and frequent visits to hospitals. Although the capacity limitations of hospitals necessitate delays, oncologists worldwide are using the power of the internet to reach millions of patients located in different geographic regions and minimize the harm caused by the interruption of anticancer treatment. Many of these oncologists have shared their valuable experience of the management and continuing education of their patients without COVID-19 (2–4). On May 19, 2020, ASCO issued a special report: A Guide to Cancer Care Delivery During the COVID-19 Pandemic (5). ESMO (European Society of Medical Oncology) also issued guidelines for patients and oncologists to deal with COVID-19 (6, 7). There are some special issues that should draw our attention when cancer patients encounter COVID-19. This short review aims to summarize the incidence and diagnostic difficulties of COVID-19 in cancer patients and discuss the anticancer treatment and prognosis of COVID-19-infected cancer patients.

## Methods

PubMed database was searched by using COVID-19 and SARS-CoV2 as the main terms to identify papers published through April 26, 2020 and the available clinical data was summarized in Table 1.

**Table 1:** Clinical data reporting the infection rate, severe illness rate and mortality of COVID-19-infected cancer patients.

**Author**	**Location**	**Single center**	**Time interval**	**Total COVID-19 patients (a)**	**COVID-19-infected cancer patients (b)**	**b/a (Infection rate of cancer patients)**	**Severe/critical rate of COVID-19 cancer patients^c^**	**Mortality of cancer patients^d^**	**Reference**
Guan W	China	NO	−1.29, 2020	1,099	10	0.9%	30%	10%	(17)
Wu Z	China	NO	−2.11, 2020	44,672	/	/	/	5.6%	(18)
Liang W	China	NO	12.30, 2019–1.31, 2020	1,590	18	1.1%	39%	/	(10)
Jing Y	Wuhan, China	YES	12.30, 2019–2.17, 2020	/	12	0.79%**^e^**	25%	25%	(11)
Zhang L	Wuhan, China	NO	12.30, 2019–2.26, 2020	/	28	/	53.6%	28.6%	(12)
Chen J	Shanghai, China	YES	1.20, 2020–2.6, 2020	249	1	0.4%	0	0	(19)
Ma J	Wuhan, China	YES	1.1, 2020–3.30, 2020	1,380	37	2.7%	54.1%	13.5%	(9)
Zhang H	Wuhan, China	NO	1.5, 2020–2.18, 2020	1,548	67	4.3%	47.8%	26.9%	(13)
Miyashita H	New York, USA	YES	3.1, 2020–4.6, 2020	5,688	334	6%	/	11.1%	(20)
Nelson B	Park Ridge, USA	YES	−4.9, 2020	/	85	/	47.1%	/	(21)
Palmieri	Italy	NO	−4.20, 2020	3,200	/	/	/	16.1%**^f^**	(22)

c *Severe/critical rate of COVID-19 cancer patients = n (severe COVID-19 positive cancer patients)/n (total COVID-19 positive cancer patients)*;

d Mortality of COVID-19 positive cancer patients = n (death of COVID-19 positive cancer patients)/n (total COVID-19 positive cancer patients)

e *The infection rate in this research was calculated by n (COVID-19 positive cancer patients)/N (total cancer patients observed)*;

f *The mortality rate in this research was calculated by n (death of COVID-19 positive cancer patients)/n (death of diseases data available COVID-19 positive patients). /, not reported*.

## Results

### Is There an Increase in the Susceptibility, Critical Illness Rate and Mortality Due to COVID-19 in Cancer Patients?

Since cancer is associated with the overexpression of immunosuppressive cytokines, a reduction in proinflammatory danger signals, an increase in the functional immunosuppressive leukocyte population (8) and increased toxicity due to anticancer treatment, cancer patients are a potentially vulnerable group. However, are cancer patients truly more vulnerable to COVID-19? The groups in China, the USA and Italy reported that cancer patients are more likely to be infected with the COVID-19 and develop severe symptoms than the general population. The infection rate for cancer patients ranged from 0.4 to 4.3% in China and was 6% in the USA according to the current published data (Table 1). In Wuhan, the infection rate for cancer patients was 2.7% (37 of 1,380 patients), which was 6 times higher than the general population in Wuhan until Mar 30, 2020 (0.45%, 50,006 of 11,081,000) (9). As for the severe illness rate, it ranged from 0 to 54.1% in China and was 47.1% in the USA (Table 1). The mortality of COVID-19 positive cancer patients is much higher than the 6.95% mortality of the entire population (Data from COVID-19 map of John Hopkins University on April 26, 2020). The mortality ranged from 0 to 28.6% in China according to the current reports. The mortality was 11.1% in the USA and 16.1% in Italy (Table 1). In Shanghai China, since the only 1 infected cancer patients didn't develop severe illness, the severe illness rate and mortality for cancer patients in this center was 0.

Interestingly, the most common cancer type in patients differs in different studies; in some studies, the most common type was lung cancer (10–13), and in one study, the most common type was colorectal cancer (9). In addition, patients who underwent chemotherapy or surgery in the past month had a numerically increased risk (three [75%] of four patients) of clinically severe events compared to those who did not receive chemotherapy or surgery (10). However, clinical data from Ma J showed no difference between these two groups (9). Otherwise, the studies did not report the relationship between the tumor stage and the susceptibility or severe illness rate. What's more, biomarkers from peripheral blood, such as lactic dehydrogenase (LDH), lymphocyte, high-sensitivity C-reactive protein (hs-CRP) and d-dimer >1 μg/mL, and clinical features, such as older age and high Sequential Organ Failure Assessment (SOFA) score, were reported to be crucial predictive biomarkers of disease mortality (14, 15).

Ling Peng holds the view that these data are insufficient to conclude that patients with cancer have an increased risk because of the small sample sizes and high heterogeneity (16). Frequent visits to the hospital and advanced age are also high-risk factors in cancer populations. Although multicenter retrospective studies with larger sample sizes and adjustment of confounding factors are needed to determine if cancer patients have increased susceptibility and critical illness rates, we should pay greater attention to protecting cancer patients from COVID-19.

### Diagnostic Difficulties in Distinguishing Between COVID-19 and Cancer: COVID-19 or Not?

Fever and respiratory symptoms are the most common symptoms of COVID-19, which may occur more frequently in cancer patients than in the healthy population. These overlapping symptoms confound the COVID-19 diagnosis. During the SARS epidemic in 2004, 11 cancer patients who met the WHO diagnostic criteria for probable SARS were admitted to hospitals, but only 1 of them was finally confirmed as having SARS (23). For patients with lung cancers, cough and fever caused by pneumonia are common initial clinical symptoms (24). Interstitial infiltrate pneumonitis exhibited by patients with primary lung cancer (25) caused by radiotherapy and checkpoint inhibitor therapy overlaps with the symptoms and CT features of COVID-19 (26). For all cancer types, patients may develop fever because of bacterial infections after chemotherapy and surgery or tumor necrosis after TACE/ultrasound ablation. Due to these specific issues, it is difficult to determine whether cancer patients are infected by COVID-19 based on clinical symptoms and CT images. Oncologists may first perform differential diagnosis of patients online based on medical history and travel history.

In some cases, symptoms may last for days, and it is difficult to exclude COVID-19. Approximately 30% of infections in cancer patients are suspected to be due to hospital-associated transmissions (12). Cancer patients with suspected COVID have to go to the hospital for nucleic or serum testing and will be exposed to a high risk of infection. Household test kits should be developed for these special groups, who may show similar symptoms because of their primary disease, to reduce nosocomial infection. Physicians at the Gustave Roussy Cancer Institute (GRCI) set up the CAPRI telemedicine program to help COVID-19-positive cancer patients. Philippe E. Spiess proposed a simple 5-part strategy to address COVID-19 in patients with cancer (27). In brief, hospitals, local health authorities, and state and national leaders will need to work much more closely together to expand the screening test quickly to all cancer patients at high risk without pretesting criteria such as travel history and fever: the elderly, patients receiving anti-cancer therapy and advanced-stage cancer patients. What's more, they hold the view that chemotherapy and surgery should be temporarily postponed for COVID-19 positive cancer patients.

Additional contactless diagnosis and treatment procedures should be established for cancer patients to reduce nosocomial infections. The guideline for cancer patients issued by ESMO recommended an untouched food delivery and medical follow-up way to prevent infection for cancer patients and reminded them to pay attention to their mental health (6).

### Continuing Anticancer Therapy or Not in COVID-19 Infected Cancer Patients?

There is no doubt that higher priority should be given if the patient's condition is immediately life threatening or clinically unstable. For those patients who are in stable or mild condition, it remains controversial whether their anticancer therapy should be stopped. Concerning deterioration due to infection, the official French guidelines recommended that pneumonia should be treated first rather than cancer (28). During chemotherapy, cytotoxic treatments diminish lymphocyte populations temporarily, potentially making patients more susceptible to infection. The report by Liang revealed that patients who underwent chemotherapy in the past month had a numerically higher risk of clinically severe events than those who did not (10). For immune therapy, pneumonitis and cytokine release syndrome are possible side effects of immune checkpoint inhibitor therapy, which may worsen COVID-19 (29). Severe adverse events are also associated with immune checkpoint inhibitor therapy (30). A lung cancer patient treated with nivolumab, a PD-1 checkpoint inhibitor, showed a rapid worsening of their condition and died 5 days after diagnosis (31). Consequently, patients on immunotherapy could be at increased risk from COVID-19. During radiotherapy, pneumonia caused by radiotherapy in lung cancer patients was also an additional risk factor for the deterioration of lung injury. According to one survey, 86.3% of COVID-19 patients were discharged after 16 (12–20) days of hospitalization (19). A one-month interruption of anticancer treatments seems to be acceptable.

However, Ma J reported that anticancer therapy did not affect the severity of COVID-19 among cancer patients (9). Another case report showed that a 57-year-old lung cancer patient infected with mild COVID-19 continued targeted therapy with stable cancer control and recovered from pneumonia after Kaletra (lopinavir/ritonavir) treatment (32). Studies of cancer patients coinfected with another virus, such as HIV, HBV or HPV, may be encouraging. Interestingly, HIV-1 and HBV are not reactivated during chemotherapy in cancer patients (33), indicating the feasibility of continuing anticancer treatment in patients with COVID-19.

In summary, we should not underestimate the risk of a more severe course of COVID-19 in cancer patients, since it will not be known if severe pneumonia is more likely to be fatal than cancer until there is enough clinical and basic research data to support continuing anticancer treatment of cancer patients co-infected with COVID-19. The guideline issued by ESMO also recommended that treatment for COVID-19 infection should be given priority over cancer. And cancer treatment will resume once patients have recovered sufficiently from COVID-19 (6).

### Favorable or Unfavorable Impact on Cancer After Survival From COVID-19

If a cancer patient survives COVID-19, the impact of coronavirus infection on the cancer prognosis is currently unknown. The UK launched the Coronavirus Cancer Monitoring Project on March 18, 2020 (34). The project will collect data on COVID-19-positive cancer patients, including data on the tumor type, stage, age, present cancer treatment, and clinical outcomes. Here, we discuss the potential favorable and unfavorable factors impacting infected cancer patients.

In addition to infecting the lungs, the new coronavirus will attack any part of the body and cause serious harm to the human body (35). The ACE2 protein is an important target of coronavirus to infect the human body and enter cells (36) and it is overexpressed in some cancers, including cervical, pancreatic and renal carcinomas, and is expressed at low levels in breast, liver and prostate cancer (16). However, it remains unknown whether overexpression of ACE2 in solid tumors will encourage viral attack and whether the virus will promote or inhibit tumor progression or metastasis.

Solid evidence is currently lacking for an effective antiviral therapy. There are 306 ongoing registered trials for the treatment of COVID-19 as of 4 April 2020, including trials of IL-6- and IL-6-receptor (IL-6R)-blocking antibodies, as severe COVID-19 causes strong expression of chemokines and inflammatory cytokines, especially IL-6 and IL1RA (37). Sarilumab is approved for treatment of Castleman syndrome by the FDA (38). The clinical trial of sarilumab showed positive trends in the “critical” group (39). Tocilizumab was listed in the latest version of the diagnosis and treatment guidelines for COVID-19 in China (40). The IL6/JAK/STAT3 pathway was reported to promote the metastasis of colorectal cancer (41). In addition, there is an ongoing clinical trial of camrelizumab, an anti-PD-1 antibody, in China for treatment of COVID-19 (ChiCTR200002806). The application of these drugs theoretically plays a role in inhibiting specific cancers.

For COVID-19-positive cancer patients with mild disease, in addition to the impact of delayed cancer treatment, the impact of the virus on prognosis is similar to that in the healthy population. For recovered patients who had severe COVID-19, the cytokine storm and the application of several anticancer or antivirus drugs may have a complex effect on the body and tumors. At present, the effects of COVID-19 and drug treatment on tumor prognosis are unknown, and we hope the UK cancer monitoring project will be discussed in the future.

## Discussion

When the general epidemic situation is improved, an online medical treatment mode can be gradually developed for cancer patients to relieve stress and delay medical treatment. Although sufficient clinical data are lacking that indicate that cancer patients are more susceptible to COVID-19, we should pay more attention to protecting them from coronavirus. In terms of the effects of cancer itself and anticancer treatment, cancer patients are more likely to develop fever or cough than the healthy population, which increases the difficulty of COVID-19 diagnosis. We call on hospitals to set up special outpatient facilities for suspected cancer patients to reduce the risk of nosocomial infection. For the unfortunately infected cancer patients, whether to continue chemotherapy, immune therapy or radiotherapy remains controversial. Since it is difficult to predict the outcome of patients with COVID-19, the recommendation of antitumor treatment still needs to be very cautious. To better assess the impact of coronavirus infection on the prognosis of cancer patients, we hope the UK cancer monitoring program will be helpful in the future.

Thank you to all the medical staff and researchers who fought hard in this unprecedented situation. We hope that all human beings will overcome this pandemic as soon as possible and that the consequences will not be too devastating for patients.

## Data Availability Statement

The original contributions presented in the study are included in the article/supplementary material, further inquiries can be directed to the corresponding author/s.

## Author Contributions

LQ conceived and wrote this manuscript. KW collected the clinical data from published studies. CY provided inspiration for this article. SZ revised and polished the manuscript. All authors contributed to the article and approved the submitted version.

## Conflict of Interest

The authors declare that the research was conducted in the absence of any commercial or financial relationships that could be construed as a potential conflict of interest.
